# COVID-19 Outbreak Effects on Job Security and Emotional Functioning Amongst Women Living With Breast Cancer

**DOI:** 10.3389/fpsyg.2020.582014

**Published:** 2020-10-29

**Authors:** Bethany Chapman, Jessica Swainston, Elizabeth A. Grunfeld, Nazanin Derakshan

**Affiliations:** Department of Psychological Sciences, Birkbeck, University of London, London, United Kingdom

**Keywords:** breast cancer, COVID-19, anxiety, depression, cognition, employment, job security

## Abstract

The outbreak of Coronavirus disease 2019 (COVID-19) has negatively impacted global economies and employment. In the UK, it is predicted that approximately eight million jobs were furloughed as a result of the outbreak and the associated restriction of movement or shielding measures. This study aimed to investigate the impact of changes in employment status on cognitive and emotional health as well as perceptions of work. Furthermore, it examined the relationships between women’s job security and anxiety, depression and cognitive function. Women living with breast cancer (*N* = 234) completed online questionnaires to measure their cognitive function, general emotional well-being, COVID-19 related emotional vulnerability (COVID-EMV), work ability and COVID-19 related perceptions of work. Our results revealed that threat to job security was predictive of depression and cognitive function in the entire sample Such that those with higher levels of perceived job security had lower depression and better cognitive function. Further, women who were furloughed or unable to continue work reported higher job insecurity compared to those who had worked throughout the pandemic. Greater rumination was also associated with worse anxiety and depression as well as poorer cognitive function. Finally, moderation analysis highlighted that women who had better cognitive functioning were less likely to experience anxiety when their job security was high. Given our findings, we suggest that employers provide women with accessible interventions to enhance cognitive and emotional resilience and thus help protect against the detrimental effects of job insecurity created by the COVID-19 outbreak.

## Introduction

Coronavirus disease 2019 (COVID-19) has had a substantial impact on global economies and individual employment (Qualtrics, 2020). In the United Kingdom (UK), the government announced a social lockdown to reduce the spread of the virus and prevent the National Health Service (NHS) becoming overwhelmed by large numbers of COVID-19 cases (Government, UK, 2020). The lockdown included a ban on all non-essential travel as well as the closure of the majority of non-essential businesses, instructing workforces to complete their work from home (if feasible). Where working at home was not possible, companies either issued redundancies or furloughed staff under the government Job Retention Scheme. It is estimated that over eight million jobs were furloughed in the UK, during which time the Government paid up to 80% of the UK median salary, to a maximum of £2,500 (Bell et al., 2020). Recent figures, however, indicate that being furloughed by an employer is associated with poorer mental health, with higher levels of stress and anxiety recorded (Qualtrics, 2020).

The COVID-19 crisis has been a particularly concerning time for vulnerable groups of individuals living with pre-existing health conditions, including women with a breast cancer diagnosis. Whilst the advances in medical treatment and earlier diagnosis mean that a significant proportion of women now survive a diagnosis for many years (10-year survival rate is 76%; Breast Cancer Now, 2020), the longer-term consequences and sequela of breast cancer and its treatment(s) are now well-established. Indeed, up to 75% of women report post-treatment cancer-related-cognitive-impairments (CRCI) (Jansen et al., 2011; Koppelmans et al., 2012; Ganz et al., 2013; Janelsins et al., 2014; see Ahles and Root, 2018 for review) which are associated with greater levels of anxiety and depression (Kaiser et al., 2019; see Papanastasiou et al., 2019 for review). Similarly, CRCI’s have been associated with a poorer quality of life (Menning et al., 2016) and worse work ability (Calvio et al., 2010; Zeng et al., 2016).

However, being in work is considered instrumental to women’s cognitive and emotional recovery and in promoting a better quality of life (Timperi et al., 2013; Keim-Malpass et al., 2016). The beneficial effects of work on cognitive ability may occur through increasing neuroplasticity (or cognitive reserve) of the brain via consistent positive stimulation (i.e., processing of new or complex information through social interaction within the workplace), as well as by reduced levels of anxiety, depression and the lessening of financial-related stress, as a consequence of receiving a wage (Vance et al., 2016). Furthermore, being in work has significant psychological benefits including providing a sense of meaning, identity and normality for many women living with cancer (Rasmussen and Elverdam, 2008; Johnsson et al., 2010; Blinder et al., 2012).

Conversely, involuntary job loss and unemployment have consistently been shown to have a significant and long-term impact on mental health (Gallo et al., 2000). Compounding this, the emergence of depression following job loss increases the risk of continued unemployment (Stolove et al., 2017). In the same way, job insecurity is considered to be a stressor that is detrimental to well-being and mental health (Llosa et al., 2018), and is associated with increased levels of depression (Blom et al., 2015). Such adverse outcomes are of additional concern for vulnerable populations already experiencing high levels of emotional distress (anxiety and depression). Women living with breast cancer have a greater risk for developing clinical affective disorders, including long-term anxiety and/or depression, as well as elevated levels of worry (Burgess et al., 2005; Avis et al., 2015; Cvetković and Nenadović, 2016; see Carreira et al., 2018 for a review). Studies have shown that both worry and rumination are significantly associated with anxiety and depression (Nolen-Hoeksema, 2000; Beckwé et al., 2014; Ryum et al., 2017; Spinhoven et al., 2018; Brown et al., 2020). Moreover, women living with breast cancer are at a greater risk of experiencing suicidal ideations and suicide up to 25 years after their diagnosis (Schairer et al., 2006; Gaitanidis et al., 2018; see Carreira et al., 2018 for review) compared to the general population. This is of significance as it is estimated that a rise in unemployment in the general population from 4.94 to 5.64% (24.7 million job losses, worldwide) as a result of COVID-19 could be accompanied by an additional 9,570 suicides each year (Kawohl and Nordt, 2020).

In view of these existing predispositions and the value placed on work by women after diagnosis and treatment, women living with a breast cancer diagnosis are more susceptible to experiencing emotional disorders and poorer mental health outcomes as a result of the distress and trauma caused by threats to job loss and job security. As such, we aimed to examine the effects of COVID-19 generated employment status (i.e., continued working or being furloughed) on perceptions of job security, work importance and employer support in response to the pandemic. We also aimed to investigate how threats to job security would predict levels of emotional distress including anxiety and depressive related symptomatology as well as cognitive function. We predicted that there would be differences in women’s perceptions of work depending on their COVID-19 generated employment status. We also predicted that the threat and uncertainty induced by COVID-19 to employment security would be associated with worse cognitive function and increased levels of vulnerability to anxiety-related symptomatology including depression.

## Materials and Methods

### Design

A cross-sectional survey design was utilized. The study was approved by the Research Ethics Committee of the Department of Psychological Sciences, the College Research Ethics Committee at Birkbeck College, University of London, and the Economic and Social Research Council.

### Participants

Women were recruited using voluntary sampling via advertisements on social media platforms such as “Building Resilience in Breast Cancer Centre” (BRiC Centre)^1^^2^ and Breast Cancer Now^3^ during the peak of the COVID-19 outbreak in the UK. Participants completed this study between the 9th of April and 26th of May 2020. The inclusion criteria for this study were women aged 18 years or older, living with a diagnosis of breast cancer, at any stage of active treatment, hormone blocker therapy or target therapy. Women could also be employed, self-employed, undertaking voluntary work or not undertaking any work at the time of recruitment.

### Materials

#### Demographic and Clinical Questionnaire (DQ)

The self-reported DQ (developed by the authors) comprises of 29-items (i.e., grade or type of treatment), sociodemographic factors (i.e., education, ethnicity and civil status), pre-existing psychological or affective disorders and employment including employment type (i.e., employed or self-employed), collar group, employment sector and the number of hours worked (part-time or full-time).

#### Functional Assessment of Cancer Therapy-Cognitive Scale (FACT-Cog, Version 3; Wagner et al., 2009)

The 37-item Fact-Cog is widely used in breast cancer research (Von Ah and Tallman, 2015; Von Ah et al., 2018) to assess self-reported cognitive impairment (PCI measured by 20 items), cognitive ability (PCA measured by nine item questions), the impact of cognitive impairment on quality of life (QoL measured by four items) and others’ comments about cognitive impairments (CFO measured by four items). Items are measured on a five-point Likert scale from “never” or “not at all” (0) to “several times a day” or “very much” (4) with negatively phrased items (PCI, QoL, CFO) reverse scored. The total score ranges from 0 to 148, with a higher total score calculated from the summation of the four subscales indicating a better cognitive function. Current study’s Cronbach’s α = 0.97.

#### Rumination Response Scale (RRS; Treynor et al., 2003)

The self-report RRS is a highly reliable questionnaire which has been used previously in breast cancer research (Steiner et al., 2014). The RRS assesses the severity of depressive rumination experienced by an individual. All 22-item statements are measured on a Likert scale with 1 indicating “never” and 4 “almost always.” The total score from the summation of items ranges from 22 to 88, with greater scores showing a higher rumination. Current study’s Cronbach’s α = 0.94.

#### Hospital Anxiety and Depression Scale (HADS; Zigmond and Snaith, 1983)

The HADS examines the severity of anxiety and depression symptomology experienced over the last week. In particular, the HADS assesses 14 items on a Likert scale with response scores ranging from 0 to 3. Seven item statements measure anxiety and seven measure depression. Scores range from 0 to 21, with greater scores on each of the subscales showing that a worse severity of symptomology is being experienced. The HADS is widely implemented in breast cancer research (Osborne et al., 2004; Akel et al., 2017) and has been shown to adequately measure anxiety and depression in breast cancer (Hopwood et al., 1991). Current study’s Cronbach’s α = 0.89.

#### Penn State Worry Questionnaire (PSWQ; Meyer et al., 1990)

The PSWQ is widely used including, in the breast cancer population (Swainston and Derakshan, 2018) to assess the level of trait worry being experienced by an individual. All 16 self-report items are measured on a five-point Likert scale with 1 indicating that the statement behavior is “not typical of me” and 5 “very typical of me.” The total score ranges from 16 to 80, with a higher total score indicating a worse level of pathological worry. Current study’s Cronbach’s α = 0.94.

#### Modified Self-Report-Generated Charlson Comorbidity (CCI; Charlson et al., 1987)

The modified CCI assesses health comorbidities (i.e., “heart trouble” and “kidney disease”) experienced by an individual. Each of the 9 comorbidities [(1) Asthma, emphysema or chronic bronchitis, (2) Arthritis or rheumatism, (3) Diabetes, (4) Digestive problems, (5) Heart trouble, (6) HIV illness or AIDS, (7) Kidney disease, (8) Liver problems, (9) stroke] included in the questionnaire is weighted (value of 1, 2, 3, or 6) with a more severe comorbidity given a higher value (i.e., “HIV illness or AIDS” has a weighted value of 6). Items are summed together to form the overall score. Greater scores show worse comorbidity. The CCI has previously been used in breast cancer research (Fu et al., 2015).

#### Work Limitations Questionnaire (WLQ; Lerner et al., 2001, 2003)

The WLQ is a self-report inventory composed of 25 positively or negatively phrased items that measure how health-related condition(s) such as breast cancer impact everyday workplace performance and productivity (Von Ah et al., 2018). Items are divided into four sub-domains (Time Management Scale, Physical Demands Scale, Mental/Interpersonal Demands Scale and the Output Demands Scale) and measured on a five-point Likert scale from 1 [“difficult all of the time (100%)” or “able all of the time (100%)”] to 5 [“difficult none of the time (0%)” or “able none of the time (0%)”] with reverse scoring for subscales 1, 3, and 4. The percentage of work productivity loss in the workplace over the last 2 weeks is calculated from the four subscale total scores. Each of the four sub-domains has a scoring range from 0 to 100 after conversion, with higher scores indicating a greater level of difficulty in the workplace. Applying the exponential formula, a percentage score for work productivity loss is calculated (the maximum attainable score for work productivity loss is 24.9%), a higher score represents a greater loss of work productivity. Current study’s Cronbach’s α = 0.97.

#### COVID-19 Items

The COVID-19 items (developed by the authors) assess the impact of the COVID-19 pandemic and lockdown on women living with breast cancer. There are two subsections, the first (comprising of 16 items) includes individual items exploring the effects of the pandemic on women’s emotional vulnerability. Specifically, these items ask participants to reflect on whether the outbreak has made them feel more (1) anxious, (2) upset, (3) fearful or less (4) in control, and (6) confident. A reliable composite score from these five items was created (Cronbach’s α = 0.89) and referred to as COVID-EMV. Items examining the COVID-19 symptoms experienced during the pandemic (i.e., fever, cough, shortness of breath, chest pain or pressure, sore throat, sneezing or runny nose, loss of smell or taste), self-isolation status, disruption to oncology appointments, receipt of the UK Government shielding letter as well as the concerns associated with the restrictive measures imposed by the shielding letter were developed.

The second subsection comprised of eight individual items that assess the impact of COVID-19 outbreak on women’s current work status (i.e., working or not work due to COVID-19) and asks participants to reflect on how COVID-19 has changed or impacted their views of work (“has the COVID-19 outbreak changed your view on the importance of your work?,” “how has the COVID-19 outbreak impacted your job satisfaction?” “has the COVID-19 outbreak changed how confident you feel at work?,” “has the COVID-19 outbreak changed your view on how secure your job is?”). Employer’s support in response to the pandemic is also assessed (“please rate your employer’s support in response to the COVID-19 impact on work”). All items are measured on a Likert scale ranging from “much less” or “not at all” (0) to “much more” or “extremely” (5), with higher scores indicate more positive views of work and better employer support. Cronbach’s α = 0.74. Individual work items were used in our analysis.

### Procedure

Women who responded (by email) to one of the study advertisements on social media platforms were sent a return email containing the study information and a secure URL to access the questionnaire. Participants were asked to provide online consent before completing the battery of online questionnaires. All participants were instructed to complete the DQ, the general cognitive and emotional health questionnaires and the main COVID-19 subsection. Participants who reported that they were employed, self-employed or who undertook volunteering work were asked to additionally complete the COVID-19 work subsection and the WLQ. A £5 gift voucher was emailed to all participants upon completion.

### Statistical Analysis

Data analyses were conducted using the Statistical Package for the Social Sciences (SPSS, version 25). Participant clinical, sociodemographic and work characteristics were explored with descriptive statistics (see Table 1).

**TABLE 1:** Clinical, sociodemographic, and work characteristics.

	***N* = 234 (%)**
** Sociodemographic**	
Age	Mean = 51 Years (Min = 27, Max = 78)
Education	
Secondary education	26 (11.1)
Further education	50 (21.4)
Higher education	152 (65.0)
Other	6 (2.6)
Ethnicity^a^	
White	222 (94.9)
Black	3 (1.3)
Asian	5 (2.1)
Multi-ethnic	3 (1.3)
Civil status^b^	
Married/Civil partnership/Cohabiting	173 (73.9)
Divorced/Separated	19 (8.1)
Single/Widowed	38 (16.2)
**Work**	
Employment status	
Employed	147 (62.8)
Self-employed	25 (10.7)
Undertaking volunteering work	14 (6.0)
Not undertaking any form of work	48 (20.5)
Collar group^c^	
White collar	117 (65.7)
Pink collar	51 (28.7)
Blue collar	3 (1.7)
Other	7 (3.9)
**Clinical—Breast cancer history**	
Age at diagnosis	Mean = 47 Years (Min = 24, Max = 77)
Time since diagnosis	Mean = 51.46 Months (Min = 0, Max = 177)
Grade^c^	
Grade 1	28 (12.0)
Grade 2	86 (36.8)
Grade 3	117 (50.0)
Active treatment	
Yes	15 (6.4)
No	215 (91.9)
Due to start	2 (0.9)
Other	2 (0.9)
Type of treatment received^d^	
Chemotherapy	171 (73.1)
Radiotherapy	186 (79.5)
Surgery	
Mastectomy	97 (41.5)
Lumpectomy	98 (41.9)
Mastectomy and Lumpectomy	23 (9.8)
Endocrine therapy	
Yes	161 (68.8)
No	63 (26.9)
Other (i.e., Prescribed but decided not to take it)	10 (4.3)
Time since treatment finished	Mean = 38 Months (Min = 0, Max = 140)
History of psychological disorders	100 (42.7)
Prescribed medication for conditions other than cancer	49 (20.9)

*^*a*^One participant did not report their ethnicity. ^*b*^Four participants did not state their current civil status. ^*c*^Fifty-six participants (including those not undertaking any form of work) did not report their collar group. ^*d*^Three participants did not report their breast cancer grade. ^*e*^One participant did not report the type of treatment received.*

One-way ANOVA’s were used to investigate the impact of employment status (i.e., working, not working due to COVID-19 or never working) on cognitive function, general anxiety and depression as well as COVID-19 related emotional vulnerability (COVID-EMV). Partial eta squared effect sizes were calculated. In addition, two independent *t*-tests were performed to examine the effects of employment type (employed vs. self-employed) on women’s general levels of anxiety and depression during this outbreak. Furthermore, independent *t*-tests were conducted to explore the effects of COVID-19 generated work status (i.e., continued working or furloughed) on employer support, the importance of work and job security. Cohen’s *d* effect sizes were calculated. *Post-hoc* analyses were conducted using G^∗^Power software (Faul et al., 2007, 2009).

Hierarchical regression analyses were run to examine the relationship of women’s job security to four dependent variables including, cognitive function, anxiety and depression as well as emotional distress after allowing for clinical and sociodemographic predictors as well as employment type (i.e., employed, self-employed or volunteering). On the first step, (1) education level, (2) age at diagnosis, (3) time since diagnosis (in months), (4) treatment status, (5) grade of breast cancer, (6) pre-existing co-morbidities (assessed by the CCI) and (7) employment type were added. Women’s rumination, worry and COVID-19-EMV were entered on step two. Finally, job security was included in the third step. Cohen’s *f*^2^ effect sizes were calculated for each of the regressions. Assessing standardized residuals, no outliers were identified in the four regression analyses: anxiety (std Residual Min = −2.4, std Residual Max = 2.6), depression (std Residual Min = −2.3, std Residual Max = 2.9), emotional distress (std Residual Min = −2.2, std Residual Max = 2.7) and cognitive function (std Residual Min = −2.6, std Residual Max = 2.4). In addition, no violations of the assumptions of collinearity, independent error, normality, homoscedasticity and linearity were found. *Post-hoc* achieved power calculations were carried out with G^∗^Power software (Faul et al., 2007, 2009) using Cohen *f*^2^ and a significance of 0.05.

Finally, moderation analyses were conducted to explore the moderating role of cognitive function on job security in predicting general anxiety and depression. Self-reported cognitive function and job security were mean-centered prior to analyses. Checks for violations of the assumption of heteroscedasticity were performed and all standard errors in the model were based on the Heteroscedasticity Consistent Standard Error (HC1).

There was no missing questionnaire data for the FACT-Cog, HADS, RRS, PSWQ, and CCI in the present study. Scores for the four sub-scales of the WLQ (time management demands, physical demands, mental/interpersonal demands and work output demands) were calculated if half or more of the scale’s questions had been answered by dividing the sum of the answered questions by the number of answered questions and then converted into a final scale score. Missing data in the WLQ was likely due to the COVID-19 induced work changes. Only 13 participants who were employed but furloughed or unable to work as a result of the COVID-19 outbreak failed to complete the individual COVID-19 work items. These participants were excluded from any analysis examining these items as scale and person-specific means were unable to be computed and substituted for the missing items.

## Results

### Sample Description

Women recruited to participate in the current study (*N* = 234) were between the age of 27 and 78 years old (*Mean* = 51 years; see Table 1 for participant clinical, sociodemographic and work information) and had a diagnosis of primary breast cancer. Of the 234 women who participated, 35 (15%) stated that they had experienced COVID-19 symptoms (4.7% high fever, 5.5% cough) although no cases had been officially diagnosed, and no hospital admissions were reported. Most of the women reported that they were either employed (147, 62.8%), self-employed (25, 10.7%) or completed volunteering work (14, 6.0%) prior to the outbreak of COVID-19. As a result of the outbreak, 50 (21.41%) participants stated they were no longer working or had been furloughed by their employer, whilst 127 (54.3%) had continued to work, but with appropriate adaptations to match the restrictive or protective measures implemented by the UK Government. Women who had continued to work showed a work productivity loss of 7.9% (measured by the WLQ).

### Impact of Employment on General Emotional Vulnerability and Cognitive Function

A series of one-way ANOVA’s were performed to examine the effect of employment status (i.e., continued working, not working as a result of COVID-19 or not working even prior to the outbreak) on women’s general anxiety and depression as well as their cognitive function and COVID-EMV. Results showed that there was a non-significant effect of employment status on general anxiety (*F* < 1, *ns*), depression [*F*(2, 231) = 1.4, *ns, 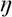
*^2^ *partial* = 0.01] and cognitive function [*F*(2, 231) = 1.6, *ns, 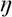
*^2^ *partial* = 0.01]. However, there was a trend towards significance for COVID-EMV [Working *Mean* = 14.6, *SD* = 6.9; Furloughed or unable work *Mean* = 13.0, *SD* = 5.6; Never working *Mean* = 15.8, *SD* = 6.5, *F*(2, 231) = 2.6, *p* = 0.08, *
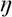
*^2^ *partial* = 0.02]. *Post-hoc* analyses showed that the achieved statistical power (1-ß err prob) was greater than 0.95 for all one-way ANOVAs performed. Moreover, independent *t*-tests examining the effects of employment type showed that there were no significant differences in the level of general anxiety and depression (*t* < 1, *ns*) reported by employed or self-employed women living with breast cancer (see Table 2 for descriptive statistics).

**TABLE 2:** Means and standard deviations for symptomology measured.

	**Employed**		**Self-Employed**	
	**M**	**SD**	**M**	**SD**
Anxiety	9.4	4.7	9.5	4.1
Depression	6.7	4.0	6.9	4.2
Cognitive function	88.5	29.4	86.5	26.6
Rumination	47.6	14.4	44.2	14.6
Worry	52.1	14.6	51.2	17.1
Health anxiety	17.8	6.9	18.6	6.8

### Impact of COVID-19 Generated Work Status on Perceptions of Work

There was a significant difference in the view of the importance of work (*t* (54.3) = 2.0, *p* = 0.05, *d* = 0.4) between women who “continued” to work (*M* = 2.8, *SD* = 1.4) during the COVID-19 outbreak and those who had been furloughed or unable to work (*M* = 2.3, *SD* = 1.5). Women who “continued” to work reported having a higher view of the importance of work. Similarly, there was a significant difference [*t*(54.4) = 3.4, p < 0.01, *d* = 0.6] found for the level of job security, with women who “continued” to work (*M* = 2.5, *SD* = 1.4) reporting a greater job security compared to those unable to work or furloughed *(M* = 1.5, *SD* = 1.6) as a consequence of the COVID-19 outbreak.

### Impact of COVID-19 Induced Job Security on General Emotional and Cognitive Function

#### Depression

The first regression analysis (Table 3) showed that when the clinical, sociodemographic, and employment type predictors were entered on step one, they accounted for a modest 5.1% of the variance in depression. After measures of worry, rumination and COVID-EMV were added on step two, the model explained an additional 35.9% [R^2^(change) = 0.359, *F*(3, 158) = 32.1, *p* < 0.001] of the variance with both rumination (*p* < 0.001) and COVID-EMV (*p* < 0.05) acting as significant predictors. On the third step, job security significantly predicted depression [*t*(157) = 2.2, *p* = 0.03] after allowing for the effects of the other predictors. A higher level of job security met with a lower level of depression in women. Cohen’s *f*^2^ = 0.63 and achieved statistical power (1-ß err prob) = 0.99.

**TABLE 3:** Hierarchical regression for the predictors of depression.

	***b***	***SE B***	**β**	***t***	***p***
**General depression**					
**Step 1**					
Constant	12.58(5.32,19.85)	3.68		3.42	0.00
Education	−0.41(−1.34,0.53)	0.47	–0.07	–0.86	0.39
Grade	0.45(−0.47,1.37)	0.47	0.08	0.96	0.34
Active treatment status	−0.68(−2.78,1.42)	1.06	–0.05	–0.64	0.52
Age at diagnosis	−0.06(−0.15,0.03)	0.05	–0.10	–1.23	0.22
Time since diagnosis (months)	−0.02(−0.04,0.01)	0.01	–0.13	–1.51	0.13
Charlson co-morbidity index	−0.11(−0.92,0.70)	0.41	–0.02	–0.27	0.79
Employment type	−0.62(−1.73,0.49)	0.56	–0.09	–1.10	0.27
**Step 2**					
Constant	1.59(−4.78,7.96)	3.22		0.49	0.62
Education	−0.04(−0.80,0.71)	0.38	–0.01	–0.11	0.91
Grade	0.15(−0.59,0.89)	0.38	0.03	0.4	0.69
Active treatment status	−1.32(−3.02,0.39)	0.87	–0.10	–1.52	0.13
Age at diagnosis	−0.02(−0.09,0.05)	0.04	–0.04	–0.53	0.60
Time since diagnosis (months)	0.01(−0.01,0.02)	0.01	0.04	0.63	0.53
Charlson co-morbidity index	0.21(−0.44,0.86)	0.33	0.04	0.63	0.53
Employment type	−0.36(−1.25,0.53)	0.45	–0.05	–0.8	0.43
Pathological worry	0.02(−0.03,0.06)	0.02	0.06	0.69	0.49
Rumination (RRS)	0.12(0.08,0.17)	0.02	0.44	5.39	0.00
COVID-EMV	0.13(0.03,0.24)	0.05	0.22	2.50	0.01
**Step 3**					
Constant	2.54(−3.81,8.89)	3.21		0.79	0.43
Education	−0.01(−0.76,0.74)	0.38	0.00	–0.03	0.98
Grade	0.20(−0.53,0.93)	0.37	0.03	0.54	0.59
Active treatment status	−1.30(−2.99,0.39)	0.86	–0.10	–1.52	0.13
Age at diagnosis	−0.02(−0.09,0.05)	0.04	–0.04	–0.57	0.57
Time since diagnosis (months)	0.01(−0.01,0.02)	0.01	0.06	0.83	0.41
Charlson co-morbidity index	0.19(−0.46,0.83)	0.33	0.04	0.57	0.57
Employment type	−0.45(−1.33,0.43)	0.45	–0.07	–1.00	0.32
Pathological worry	0.01(−0.03,0.06)	0.02	0.05	0.60	0.55
Rumination (RRS)	0.12(0.08,0.16)	0.02	0.43	5.37	0.00
COVID-EMV	0.13(0.02,0.23)	0.05	0.21	2.40	0.02
Job security	−0.36(−0.67,−0.04)	0.16	–0.14	–2.20	0.03

*95% confidence intervals.*

#### Anxiety

The results from our second regression analysis (Table 4) disclosed that the seven demographic variables included in step one accounted for 6.6% of the variance in anxiety scores reported. When worry, rumination and COVID-EMV were entered on the second step, an additional 59.5% of the variance was explained [R^2^(change) = 0.595, *F*(3, 158) = 92.4, *p* < 0.001] and all three functioned as significant predictors for levels of anxiety (*p* < 0.001). On the final step, women’s job security fell short of explaining anxiety [*t*(157) = 1.4, *p* = 0.18]. Cohen’s *f*^2^ = 1.78 and achieved statistical power (1-ß err prob) = 0.99.

**TABLE 4:** Hierarchical regression for the predictors of anxiety.

	***b***	***SE B***	**β**	***t***	***p***
**Anxiety**					
**Step 1**					
Constant	16.31(8.08,24.54)	4.17		3.91	0.00
Education	−1.09(−2.15,−0.03)	0.54	–0.16	–2.03	0.04
Grade	−0.15(−1.19,0.90)	0.53	–0.02	–0.28	0.78
Active treatment status	1.40(−0.98,3.78)	1.21	0.09	1.16	0.25
Age at diagnosis	−0.08(−0.19,0.02)	0.05	–0.13	–1.56	0.12
Time since diagnosis (months)	−0.02(−0.04,0.00)	0.01	–0.13	–1.63	0.11
Charlson co-morbidity index	−0.36(−1.28,0.56)	0.47	–0.06	–0.78	0.44
Employment type	−0.33(−1.58,0.56)	0.64	–0.04	–0.51	0.61
**Step 2**					
Constant	−2.40(−7.91,3.12)	2.79		–0.86	0.39
Education	−0.38(−1.03,0.27)	0.33	–0.06	–1.15	0.25
Grade	−0.40(−1.04,0.25)	0.33	–0.06	–1.22	0.23
Active treatment status	1.03(−0.45,2.51)	0.75	0.07	1.38	0.17
Age at diagnosis	−0.02(−0.09,0.04)	0.03	–0.03	–0.67	0.51
Time since diagnosis (months)	0.01(−0.01,0.02)	0.01	0.06	1.08	0.28
Charlson co-morbidity index	0.21(−0.36,0.77)	0.29	0.04	0.73	0.47
Employment type	0.16(−0.61,0.93)	0.39	0.02	0.41	0.69
Pathological worry	0.09(0.05,0.13)	0.02	0.30	4.61	0.00
Rumination (RRS)	0.09(0.05,0.13)	0.02	0.28	4.52	0.00
COVID-EMV	0.26(0.17,0.35)	0.05	0.38	5.66	0.00
**Step 3**					
Constant	−1.89(−7.44,3.66)	2.81		–0.67	0.50
Education	−0.36(−1.01,0.29)	0.33	–0.05	–1.10	0.28
Grade	−0.37(−1.01,0.27)	0.32	–0.05	–1.13	0.26
Active treatment status	1.04(−0.43,2.52)	0.75	0.07	1.40	0.17
Age at diagnosis	−0.02(−0.09,0.04)	0.03	–0.04	–0.69	0.49
Time since diagnosis (months)	0.01(−0.01,0.02)	0.01	0.06	1.20	0.23
Charlson co-morbidity index	0.20(−0.37,0.76)	0.29	0.03	0.68	0.50
Employment type	0.11(−0.66,0.88)	0.39	0.01	0.28	0.78
Pathological worry	0.09(0.05,0.12)	0.02	0.29	4.56	0.00
Rumination (RRS)	0.09(0.05,0.13)	0.02	0.28	4.48	0.00
COVID-EMV	0.26(0.17,0.35)	0.05	0.38	5.59	0.00
Job security	−0.19(−0.47,0.09)	0.14	–0.06	–1.35	0.18

*95% confidence intervals.*

#### Emotional Distress

The results from our third regression analysis (Table 5) showed that when the clinical, sociodemographic and employment type predictors were entered on step one, they accounted for a modest 6.1% of the variance in emotional distress (as measured by the HADS-total). After measures of worry, rumination and COVID-EMV were added on step two, the model explained an additional 57.8% [R^2^(change) = 0.578, *F*(3, 158) = 84.5, *p* < 0.001] of the variance with rumination, worry and COVID-EMV (*p* < 0.05) acting as significant predictors. On the third step, job security significantly predicted emotional distress [*t*(157) = 2.2, *p* = 0.03] after allowing for the effects of the other predictors. A higher level of job security met with a lower level of emotional distress in women. Cohen’s *f*^2^ = 1.66 and achieved statistical power (1-ß err prob) = 0.99.

**TABLE 5:** Hierarchical regression for the predictors of emotional distress (as measured by the HADS-total).

	***b***	***SE B***	**β**	***t***	***p***
**Emotional distress**					
**Step 1**					
Constant	29.13(15.18,43.07)	7.06		4.13	0.00
Education	−1.47(−3.27,0.32)	0.91	–0.13	–1.62	0.11
Grade	0.18(−1.59,1.95)	0.90	0.02	0.20	0.84
Active treatment status	0.76(−3.27,4.79)	2.04	0.03	0.37	0.71
Age at diagnosis	−0.14(−0.31,0.04)	0.09	–0.13	–1.55	0.12
Time since diagnosis (months)	−0.04(−0.08,0.00)	0.02	–0.15	–1.81	0.07
Charlson co-morbidity index	−0.50(−2.06,1.06)	0.79	–0.05	–0.64	0.52
Employment type	−0.99(−3.12,1.13)	1.08	–0.07	–0.92	0.36
**Step 2**					
Constant	−0.61(−10.21,8.99)	4.86		–0.13	0.90
Education	−0.39(−1.53,0.75)	0.58	–0.03	–0.67	0.50
Grade	−0.37(−1.49,0.75)	0.57	–0.03	–0.65	0.52
Active treatment status	−0.22(−2.80,2.35)	1.31	–0.01	–0.17	0.86
Age at diagnosis	−0.04(−0.15,0.07)	0.06	–0.04	–0.71	0.48
Time since diagnosis (months)	0.01(−0.01,0.04)	0.01	0.05	1.00	0.32
Charlson co-morbidity index	3.96(−0.59,1.38)	0.50	0.04	0.79	0.43
Employment type	−0.26(−1.60,1.08)	0.68	–0.02	–0.39	0.70
Pathological worry	0.10(0.03,0.16)	0.03	0.19	2.94	0.00
Rumination (RRS)	0.21(0.14,0.28)	0.03	0.39	6.19	0.00
COVID-EMV	0.41(0.25,0.57)	0.08	0.35	5.08	0.00
**Step 3**					
Constant	0.84(−8.73,10.41)	4.85		0.17	0.86
Education	−0.34(−1.47,0.78)	0.57	–0.03	–0.60	0.55
Grade	−0.29(−1.40,0.81)	0.56	–0.03	–0.52	0.60
Active treatment status	−0.20(−2.75,2.35)	1.29	–0.01	–0.16	0.88
Age at diagnosis	−0.04(−0.15,0.07)	0.06	–0.04	–0.76	0.45
Time since diagnosis (months)	0.02(−0.01,0.04)	0.01	0.06	1.20	0.23
Charlson co-morbidity index	0.36(−0.61,1.33)	0.49	0.04	0.73	0.47
Employment type	−0.39(−1.73,0.93)	0.67	–0.03	–0.59	0.56
Pathological worry	0.09(0.03,0.16)	0.03	0.19	2.88	0.01
Rumination (RRS)	0.21(0.14,0.27)	0.03	0.39	6.19	0.00
COVID-EMV	0.40(0.24,0.56)	0.08	0.34	5.01	0.00
Job security	−0.54(−1.03,−0.06)	0.24	–0.11	–2.24	0.03

*95% confidence intervals.*

#### Cognitive Function

Our fourth regression analysis (Table 6) revealed that the seven demographic variables added in step one account for 4.5% of the variance in cognitive function scores. After worry, rumination and COVID-EMV were entered on step two, the explained variance increased by 23.2% [R^2^(change) = 0.23, *F*(3, 158) = 16.9, *p* < 0.001] with both rumination and COVID-EMV acting as significant predictors (*p*. < 0.05). On step three, job security was a significant predictor of cognitive function [*t*(157) = 2.2, *p* = 0.03] after allowing for the effects of the clinical, sociodemographic and employment type predictors. Higher job security was associated with better cognitive function. Cohen’s *f*^2^ = 0.33 and achieved statistical power (1-ß err prob) = 0.99.

**TABLE 6:** Hierarchical regression for the predictors of cognitive function.

	***b***	***SE B***	**β**	***t***	***p***
**Cognitive function**					
**Step 1**					
Constant	83.56(31.79,135.33)	26.22		3.19	0.00
Education	2.88(−3.79,9.55)	3.38	0.07	0.85	0.40
Grade	−4.63(−11.19,1.94)	3.32	–0.11	–1.39	0.17
Active treatment status	−9.93(−24.91,5.04)	7.58	–0.11	–1.31	0.19
Age at diagnosis	0.50(−0.16,1.15)	0.33	0.13	1.50	0.14
Time since diagnosis (months)	0.10(−0.05,0.25)	0.07	0.11	1.36	0.18
Charlson co-morbidity index	−1.67(−7.45,4.12)	2.93	–0.05	–0.57	0.57
Employment type	−0.93(−8.82,6.95)	3.99	–0.02	–0.23	0.82
**Step 2**					
Constant	140.11(90.03,190.19)	25.35		5.53	0.00
Education	0.94(−5.00,6.88)	3.01	0.02	0.31	0.76
Grade	−2.85(−8.67,2.98)	2.95	–0.07	–0.97	0.34
Active treatment status	−6.54(−19.98,6.90)	6.81	–0.07	–0.96	0.34
Age at diagnosis	0.30(−0.27,0.88)	0.29	0.08	1.04	0.30
Time since diagnosis (months)	−0.03(−0.17,0.10)	0.07	–0.03	–0.44	0.66
Charlson co-morbidity index	−3.49(−8.63,1.65)	2.60	–0.10	–1.34	0.18
Employment type	−1.88(−8.87,5.12)	3.54	–0.04	–0.53	0.60
Pathological worry	0.12(−0.22,0.46)	0.17	0.07	0.71	0.48
Rumination (RRS)	−0.74(−1.09,−0.40)	0.18	–0.38	–4.21	0.00
COVID-EMV	−1.01(−1.84,−0.18)	0.42	–0.24	–2.40	0.02
**Step 3**					
Constant	132.81(82.85,182.77)	25.29		5.25	0.00
Education	0.69(−5.18,6.57)	2.97	0.02	0.23	0.82
Grade	−3.24(−9.00,2.53)	2.92	–0.08	–1.11	0.27
Active treatment status	−6.65(−19.94,6.64)	6.73	–0.07	–0.99	0.32
Age at diagnosis	0.31(−0.26,0.88)	0.29	0.08	1.08	0.28
Time since diagnosis (months)	−0.04(−0.18,0.09)	0.07	–0.05	–0.63	0.53
Charlson co-morbidity index	−3.31(−8.40,1.77)	2.57	–0.09	–1.29	0.20
Employment type	−1.19(−8.13,5.75)	3.52	–0.02	–0.34	0.74
Pathological worry	0.14(−0.20,0.48)	0.17	0.08	0.81	0.42
Rumination (RRS)	−0.73(−1.08,−0.39)	0.18	–0.37	–4.18	0.00
COVID-EMV	−0.96(−1.78,−0.13)	0.42	–0.22	–2.30	0.02
Job security	2.74(0.23,5.25)	1.27	0.15	2.16	0.03

*95% confidence intervals.*

Checks for violation of assumptions showed that the assumption of collinearity (Tolerance > 0.1, VIF < 10), independent error (Depression Durbin-Watson = 2.0; Anxiety Durbin-Watson = 1.9; Emotional Distress Durbin-Watson = 2.0; Cognitive Function Durbin-Watson = 2.1), normality and homogeneity of variance and were met for all four regression analyses performed.

### Moderating Role of Cognitive Function in the Relationship Between Job Security and Emotional Symptomatology

**FIGURE 1:**
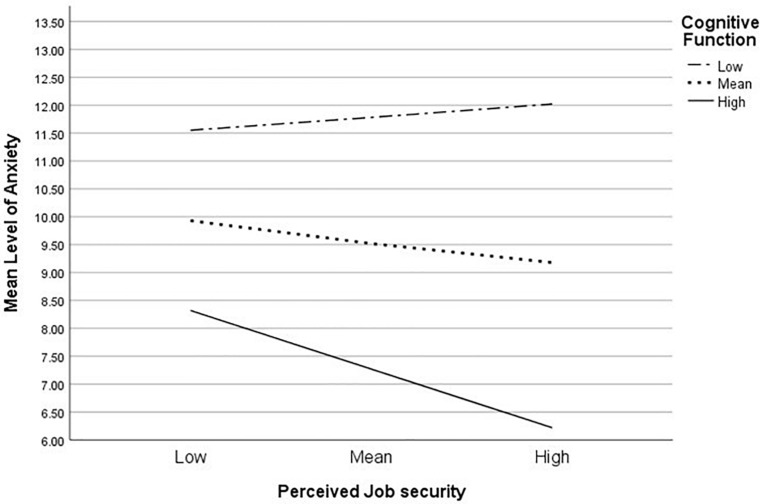
Simple slope equations for the regression of anxiety on job security at three levels of cognitive function.

Our analyses showed that cognitive function significantly moderated the relationship between job security and anxiety (*b* = −0.01, 95% CI [-0.03, -0.00], *t* = 2.2, *p* = 0.03). With higher levels of cognitive function, job security was met with lower levels of anxiety (*b* = −0.7, 95% CI [-1.1, -0.2], *t* = 2.9, *p* < 0.01), indicating that the relationship between job security and anxiety was affected by cognitive function (see Figure 1). In particular, women with better cognitive functioning and high job security reported lower levels of anxiety. There was no significant moderation found for depression.

## Discussion

To our knowledge, this is the first study to examine the effects of COVID-19 induced job insecurity and employment status (i.e., working or furloughed) on cognitive function and emotional health (general anxiety and depression) as well as perceptions of work and employer support in women living with breast cancer. As predicted, our results revealed that there were significant differences in women’s work perceptions depending on their current work status. Women who had been furloughed, or were unable to work, as a result of the COVID-19 outbreak, reported a lower level of work importance compared to those who had “continued” to work. This suggests that the outbreak of COVID-19 provoked a re-evaluation of work importance with a more detrimental effect noted for women who had been left unable to work. It is plausible that the reduction in work importance was part of a coping mechanism used by women who had been furloughed or left unable to work.

Women who were unable to work perceived a greater level of threat or uncertainty surrounding their long-term job security. The global economic recession triggered by COVID-19 could result in 24.7 million job losses worldwide, approximately two million more than the 2008–2009 global financial crisis (International Labour Organization, 2020). Given that unplanned loss of employment is associated with worse mental health outcomes (Gallo et al., 2000), and that women living with breast cancer have a pre-existing vulnerability for developing clinical affective disorders (Burgess et al., 2005; Avis et al., 2015; Cvetković and Nenadović, 2016; Carreira et al., 2018), this finding has important implications. In particular, we advocate that women furloughed by employers or who are unable to work as a result of the pandemic would likely benefit from the early implementation of interventions and support services that improve emotional and psychological resilience. Emotional distress in breast cancer survivors has been associated with poorer quality of life (Zeng et al., 2016), lower cognitive function (Von Ah and Tallman, 2015) and a high level of fatigue (Vardy et al., 2019), as well as reduced adherence to crucial treatment and medications, increasing the risk of mortality (see Theofilou and Panagiotaki, 2012 for a review). Studies show that higher levels of depression, fatigue and poorer cognitive function are in turn linked to worse work productivity and output (Calvio et al., 2010; Zeng et al., 2016; Von Ah et al., 2018). It is important that women who have been left unable to work or furloughed as a result of the COVID-19 outbreak have a high emotional resilience on their return to work as this will likely improve their work efficiency, and potentially reduce the risk of them being selected for redundancy against other candidates.

Compounding this, findings further showed that job insecurity was a significant predictor for greater levels of depression and poorer cognitive function across the entire sample. Previous research has shown that threat to job security is significantly associated with increased depressive symptoms including, loss of interest, lack of energy and lower mood (Blom et al., 2015). Similarly, we found rumination and COVID-EMV to be significant predictors of depression and cognitive function (Nolen-Hoeksema, 2000; Beckwé et al., 2014; Spinhoven et al., 2018). Such findings suggest that experiencing job insecurity exacerbates pre-existing cognitive and emotional vulnerabilities (depression) commonly reported by women diagnosed with breast cancer.

Although it has been reported previously that threat to job security in nurses is associated with increased depression *and* anxiety (Boya et al., 2008), our study found no association with anxiety. However, in line with previous studies, our findings showed that both worry and rumination were significantly associated with anxiety (Nolen-Hoeksema, 2000; Ryum et al., 2017; Brown et al., 2020). Similarly, women’s COVID-EMV was a significant predictor. One possible explanation for our non-significant finding is that the anxiety experienced by women living with breast cancer during the peak of the COVID-19 pandemic, when our study was conducted, was associated more with persistent negative thinking and fear of the possible implications if they were to catch this novel virus (e.g., high risk of health complications and premature mortality), as opposed to job insecurity. It is important that we continue to assess the effects of threat to job security and possible COVID-19 related job loss on anxiety after the peak of the COVID-19 outbreak and the lifting of restrictive measures. This will provide us with a greater insight into the specific factors triggering the symptoms of anxiety in women living with breast cancer.

Of focal importance, we identified that cognitive function moderated the relationship between job security and anxiety. That is, women with better cognitive function were less vulnerable to anxiety when job security was less of a concern. Previous studies indicate that cognitive function has a protective effect in attenuating emotional vulnerability (anxiety and depression) in women living with breast cancer. A large cross-sectional study showed that self-reported cognitive function was significantly associated with emotional vulnerability such that a better cognitive function was coupled with greater emotional well-being (Chapman et al., 2019). In addition, an intervention study conducted by Swainston and Derakshan (2018) revealed that women who received adaptive cognitive training (i.e., dual n-back training) reported less anxiety symptomology compared to the active control group. The moderating effect of cognitive function found in this study further corroborates the notion that in women living with a diagnosis of breast cancer, cognitive ability protects against the development of severe emotional symptomologies including, anxiety. Our findings suggest that women with lower cognitive function may benefit more from adaptive cognitive training interventions that improve cognitive efficiency particularly when there is a threat to their job security.

Collectively, our results suggest that the impact of the COVID-19 outbreak on work and employment (e.g., increased job insecurity) risks women living with breast cancer being susceptible to developing affective disorders and poorer cognitive function. Based on our findings we recommend that both employers and the UK Government consider providing more accessible support to ameliorate emotional and cognitive health. In particular, we suggest the remote implementation of therapies such as positive psychotherapy (Ochoa et al., 2017) and CBT (Eichler et al., 2015; Ren et al., 2019) as well as adaptive cognitive training (Swainston and Derakshan, 2018) to reduce both anxiety and depression and promote cognitive efficiency. We also advocate that where possible; employers offer women the opportunity to vocalize their concerns about possible job insecurity. Such open discussions may alleviate distress and depression or alternatively allow better preparation in the eventually of job loss.

### Limitations

The current study presents some limitations that need to be considered when interpreting the results. Firstly, our study was cross-sectional and therefore provides only a snapshot of the experiences of the women at the time of the questionnaire and also limits explanations around cause and effect. Previous research conducted by Chapman et al. (2019) found evidence for a bi-directional relationship between self-reported cognitive function and emotional well-being in women living with a diagnosis of breast cancer. We advocate that future research conduct longitudinal studies with multiple follow-up sessions as this will provide us with vital information on the trends of how COVID-19 impacts cognitive and emotional health across the pandemic. By assessing the trends and the specific predictors associated with anxiety and depression symptoms, as well as poorer cognitive function, we could provide more targeted support and interventions and thus reduce the risk of clinical affective disorders.

A second limitation is participants were asked to self-report their demographic information including, breast cancer history and pre-existing psychological or affective disorders. In the future, we would recommend that medical records are obtained and assessed to ensure the reliability of the information reported. Finally, we recruited all of our participants using voluntary sampling via online advisements placed on social media platforms (i.e., Twitter) due to the social restrictions and shielding imposed by the UK Government during the peak of the COVID-19 outbreak. As a consequence, our sample of women may not be representative of the much wider breast cancer population. The sample was also well-educated and primarily Caucasian (95%) indicating that women living with a breast cancer diagnosis from BAME backgrounds are underrepresented in our study. We suggest that future research recruit women from multiple sources including, referral from oncologists or other health care professionals.

## Conclusion

To conclude, the current study showed that women living with breast cancer who were furloughed or unable to work as a result of the COVID-19 pandemic reported a greater level of job insecurity compared to those who had “continued” with their normal work duties. Furthermore, this is the first study to show that women working with a diagnosis of breast cancer are at an increased risk for experiencing affective disorders (anxiety and depression) and poorer cognitive function as a consequence of job insecurity created by the COVID-19 outbreak and associated restrictive measures. We suggest the implementation of accessible adaptive cognitive training interventions and supportive therapies such as CBT to ameliorate the cognitive and emotional health of women experiencing concerns about their future job security.

## Data Availability Statement

The datasets for this article are not publicly available because the participants of this study did not consent to their data being shared. Requests to access the datasets should be directed to BC, bchapm02@mail.bbk.ac.uk.

## Ethics Statement

The studies involving human participants were reviewed and approved by the Research Ethics Committee of the Department of Psychological Sciences, the College Research Ethics Committee at Birkbeck College, University of London and Economic and Social Research Council. The patients/participants provided their written informed consent to participate in this study.

## Author Contributions

BC, EG, and ND: research question, funding, study design, and analysis plan. BC and JS: preparation of data and data collection. BC: drafting initial version of manuscript and drafting final version of manuscript. All authors contributed to the statistical analysis and critical review of early and final versions of manuscript.

## Conflict of Interest

The authors declare that the research was conducted in the absence of any commercial or financial relationships that could be construed as a potential conflict of interest.

## Footnotes

1 The Birkbeck Integrative Centre for Building Resilience in Breast Cancer (BRiC) delivers interactive psycho-educational support for woman living with primary or secondary breast cancer in the UK. To date, the BRiC centre has 1,827 UK members with a diagnosis of breast cancer.

2 http://briccentre.bbk.ac.uk/

3 https://breastcancernow.org/
